# A Descriptive analysis of urine drug screen results in patients with opioid use disorder managed in a primary care setting

**DOI:** 10.1186/s13722-021-00264-4

**Published:** 2021-09-30

**Authors:** Halle G. Sobel, Jill S. Warrington, Samuel Francis-Fath, Abigail M. Crocker, Claudia A. Berger

**Affiliations:** 1 University of Vermont, Robert Larner College of Medicine, VT Burlington, USA; 2 Department of General Internal Medicine, Larner College of Medicine University of Vermont, VT Burlington, USA; 3 Department of Pathology, Larner College of Medicine University of Vermont, VT Burlington, USA; 4 South Burlington, VT Averheatlh, USA; 5 Department of Mathetmatics and Statistics College of Engineering and Mathematical Sciences, University of Vermont, VT Burlington, USA

**Keywords:** Urine drug screen, Buprenorphine-naloxone, Opioid use disorder, Medications for treatment of opioid use disorder (MOUD)

## Abstract

**Background:**

Urine drug screening (UDS) is commonly used as part of treatment for opioid use disorder (OUD), including treatment with buprenorphine-naloxone for OUD in a primary care setting. Very little is known about the value of UDS, the optimum screening frequency in general, or its specific use for buprenorphine treatment in primary care. To address this question, we thought that in a stable population receiving buprenorphine-naloxone in the primary care setting it would be useful to know how often UDS yielded expected and unexpected results.

**Methods:**

We present a descriptive analysis of UDS results in patients treated with buprenorphine-naloxone for OUD in a primary care setting over a two-year period. An unexpected test result is:

1. A negative test for buprenorphine and/or
2. A positive test for opioids, methadone, cocaine and/or heroin.

**Results:**

A total of 161 patients received care during the study period and a total of 2588 test results were analyzed from this population.

We found that 64.4% of the patient population (n = 104 patients) demonstrated both treatment adherence (as measured by buprenorphine positive test results) and no apparent unexpected test findings, as defined by negative tests for opioids, methadone, cocaine and heroin. Of the 161 patients, 20 results were positive for opioids, 5 for methadone, 39 for heroin and 2 for cocaine.

Analysis at the UDS level demonstrated that, of the 2588 test results, 38 (1.5%) results did not have buprenorphine. Of the 2588, 28 (1.1%) test results were positive for opioids, 8 (0.3%) were positive for methadone, 39 (1.5%) for cocaine and 2 (0.1%) for heroin.

**Conclusion:**

Given that the majority of patients in our study had expected urine results, it may be reasonable for less frequent urine testing in certain patients.

**Supplementary Information:**

The online version contains supplementary material available at 10.1186/s13722-021-00264-4.

## Introduction

The Substance Abuse and Mental Health Services Administration federal guidelines for opioid treatment programs mandate that programs administer adequate testing for drugs of abuse, including at least eight random drug tests yearly per patient in maintenance treatment [15]. The American Society of Addiction Medicine in their recent update recommends urine drug screening (UDS) during treatment to monitor patients for adherence to prescribed medications and use of alcohol, illicit, and controlled substances. They do not give specific guidance on the frequency of testing except to note that the frequency should be determined by several factors including stability of the patient, type of treatment, and treatment setting. [3]. Interpretation of UDS results is an important tool for clinicians [11].

A relative paucity of literature exists to provide direct guidance on testing frequency in different clinical settings [5]. A systematic literature review on the effect of and recommendations for the frequency of UDS on health outcomes for persons with opioid use disorder (OUD) who receive opioid agonist therapy found only one study meeting their inclusion criteria. They noted an overall lack of evidence for the association between frequent urine drug screening and health outcomes and an “urgent gap in research evidence underpinning an area of clinical importance.” [10]. Consensus guidelines developed by the American Society of Addiction Medicine Board of Directors recommended that UDS should be done weekly in early recovery and could be decreased to monthly in stable recovery but cited in their 2017 Consensus Statement on Appropriate Use of Drug Testing in Clinical Addiction Medicine that more research in this subject would be useful [1]. Some efficacies for UDS have been identified in the pain management setting including identification of patients misusing opioids, potential diversion and prognostic value for suicidality and overdose. This study provided initial estimates on the value of UDS frequency in the chronic pain population but acknowledged additional work was needed [4].

With the prevalence of opioid use disorder amidst the opioid epidemic, the overall costs associated with urine drug screening have been increasingly apparent to payors, patients and providers alike [13]. The Vermont Medicaid program had a cost increase from over two million dollars in urine drug screen testing in 2016 to over 14 million dollars in 2018. This time period corresponded to a tripling of members receiving UDS for opioid use disorder [17]. Further, particularly egregious cases of unnecessary testing have raised concerns about the value and clinical utility of testing [12].

As care for patients with OUD using medications for opioid use disorder (MOUD) evolves to be more patient-centered, with less stigma and more privacy, providers should be able to adjust the frequency of urine testing if it is useful for ongoing care of patients in recovery [9, 14].

Given the expense of urinary drug screens and the time this requires of patients, determining the value of this test is important [8]. A determination of how often UDS yields unexpected results might be helpful in assessing their value. The primary outcome was expected and unexpected UDS results. We performed a retrospective observational study to look at unexpected UDS results on all patients receiving buprenorphine-naloxone for opioid use disorder at adult primary care practices affiliated with the University of Vermont Medical Center (UVMMC).

## Methods

### Clinical practice setting and infrastructure

This study was performed at office-based opioid treatment practice programs within nine medical homes at the UVMMC. These medical homes included 35 providers at 5 family medicine and 4 internal medicine practices in Chittenden County, Vermont. This clinical work is supported by a broader hub-and-spoke infrastructure in Vermont. Vermont’s nationally recognized hub-and-spoke model supports patients through hubs, which are staffed with health care providers with extensive training in addiction medicine, and spokes which include one or more buprenorphine-waivered physicians or advanced practice providers, a dedicated registered nurse and a licensed drug and alcohol counselor [2]. In addition to the hub-and-spoke model, the UVMMC created a unique “super spoke” called the Addiction Treatment Program (ATP) in 2016. The ATP is a multidisciplinary clinic housed under the UVMMC Department of Psychiatry and is a unique bridge clinic in the hub- and-spoke model to provide support both to patients and their primary care providers (PCPs) who would be their spoke provider.

Patients entering the office-based opioid treatment programs (or spokes) are therefore considered more stable, generally have expected UDS results and are doing well on their current dose of buprenorphine-naloxone at the time of entry into their medical home for MOUD. Stable patients are defined as those patients who have had several expected urine drug screens in a row, are on a stable dose of buprenorphine-naloxone, typically keep scheduled appointments, and were felt to be ready for spoke care by the ATP clinic.

All providers within the UVMMC spoke system follow a standardized protocol that supports providers on patient management including specific guidance on urine drug testing. In the UVMMC protocol, urine drug tests are requested randomly ranging from weekly to monthly depending on the stability of the patient. The most stable patients are monitored with monthly urine drug tests while less stable patients are monitored weekly in the primary care setting. On a random basis, patients are notified to come in for a UDS. They have 24 h to come in to perform an observed collection.

### UDS analysis and data set

Eligible patients included any UVMMC primary care patient being prescribed buprenorphine-naloxone for opioid use disorder at their medical home who had a UDS collected between 1/19/2016–1/31/18. All collected samples were evaluated at Aspenti Health (South Burlington, VT), a reference diagnostic laboratory focused on patients with substance use disorders. Testing was a composite of immunoassay screening by enzyme-linked immunoassay (EIA) and confirmation testing by Liquid Chromatography-Tandem Mass Spectrometry. If both test methods were available for a given patient sample, the confirmation result was used in this analysis. Sample characteristics including unique identification, specimen collection date and provider as well as patient demographics such as name, age, and gender were captured.

Samples were evaluated for a range of drugs including cocaine, heroin, methadone, general opiates, oxycodone, fentanyl, tapentadol and tramadol. We did not include methamphetamines due to the low prevalence regionally of this drug in our community. Given the common findings of THC, we did not to report this drug in our findings.

Laboratory results were categorized as expected or unexpected findings. The presence of buprenorphine in the urine was characterized as an expected finding. Its absence was considered unexpected. To account for the risk of specimen tampering by intentional contamination of urine with the original drug, buprenorphine confirmation testing that demonstrated only buprenorphine with no corresponding metabolite norbuprenorphine was defined as unexpected [18]. We designated 4 unexpected categories, detection of cocaine, heroin, methadone, and other opioids. Other opioid testing included codeine, fentanyl, hydrocodone, hydromorphone, morphine, oxycodone, oxymorphone, tapentadol, and tramadol. If an analytic confirmation testing was performed, the results of the confirmation overrode the initial screening results. We also looked at the presence of benzodiazepines in the UDS. However, because we did not have the medication lists to determine if these patients were prescribed a benzodiazepine, we did not characterize a UDS positive for benzodiazepines as an unexpected result. Our analytic plan evaluated the primary outcome per patient and per test. The dataset analyzed in this study was sourced from Aspenti’s laboratory information system (LIS).

The LIS database securely records, manages, and stores test order and test result data, along with other protected health information regarding the patients whose samples have been tested by AspentiDescriptive statistical methods were employed to analyze subject characteristics, using Stata 15.0 [16].

This study was reviewed and approved by the University of Vermont Institutional Review Board.

## Results

A total of 161 patients met criteria for the study and a total of 2,588 tests were analyzed from this population of patients. The number of urine tests per patient is shown in Table 1. The patients were followed for up to 660 days with an average length of time in the study period per patient was 289.6. The gender was about equal with 77 men and 79 women and gender was not indicated for 5 patients.

**Table 1:** Demographic characteristics of the study population

Individuals	161
Total test results	2588
Age at initial visit	38 (SD 11, Median 35)
Gender at initial visit	M = 77(47.8%) F = 79(49%) Unknown 5 (3%)
Tests per patient	Average 16.1 (SD 11.1, Median 15) Range per patient 1–48
Days Followed	289.6 (SD 218.5 Median 255) Range 0–660 days
Patients with buprenorphine and no unexpected substances in the urine	104 Patients (64.4%) (N = 161)

We found that 64.4% of the patient population (n = 104 patients) demonstrated both treatment adherence (as measured by buprenorphine positive test results) and no apparent unexpected test findings, as defined by negative tests for opioids, methadone, cocaine and heroin. A total of 57 patients (35.4%) did not meet these criteria.

The Table 2 shows both unexpected results at the patient and test level. The analysis at a patient-specific level demonstrated that seven patients (4.3%) had at least one UDS that did not contain buprenorphine. Twenty patients (12.4%) had at least one UDS that contained unexpected opioids. Five patients (3.1%) had at least one UDS that contained methadone. Twenty-one patients (13%) had at least one UDS that contained cocaine and two patients (1.2%) had at least one UDS that contained heroin. When combining the unexpected substances opioids, methadone, cocaine and heroin, we found that thirty-nine of the 161 patients (24.2%) were positive for at least one of those unexpected substances. In addition, seven patients (4.3%) were positive for two types and one person was positive for three types. A total of 122 patients (75.8%) were never positive for those unexpected medications.

**Table 2:** Number of unexpected patient and UDS results (percentages are in ())

Category	No bup	+Other opioid	+Methadone	+Cocaine	+Heroin	+Any unexpected substance
Patients* 161	7 (4.34)	20 (12.4)	5 (3.1)	21 (13.0)	2 (1.2)	47** (29.2)
Urine Tests 2588	38 (1.46)	28 (1.08)	8 (0.30)	39 (1.50)	2 (0.07)	77 (2.97)

^*^The same patient and the same UDS could be positive for more than one substance and negative for buprenorphine

^**^This could be more than one unexpected substance

Analysis at the UDS level demonstrated that, of the 2588 test results, 38 (1.5%) results did not have buprenorphine. Of the 2588, 28 (1.1%) test results were positive for opioids, 8 (0.3%) were positive for methadone, 39 (1.5%) for cocaine and 2 (0.1%) for heroin.

## Discussion

Urine drug screening has become integral to monitoring patients with OUD in recovery and is a tool to help prescribers regularly assess patients for unexpected medications in the urine [7]. In addition, the UDS may be an important tool to help patients keep on track with their recovery[6]. Some evidence supports its clinical utility in the pain management setting including mitigating suicidality and overdose risk, identifying potential diversion and identifying initial misuse [4]. Nonetheless, there is little evidence on how UDS affect outcomes in patients with OUD.

Given that the majority of patients in our study had expected urine results, it may be reasonable for less frequent urine testing as a more patient-centered approach. Patients who have had several expected UDS results, are on a stable dose of buprenorphine-naloxone and keep scheduled appointments and are otherwise doing well in their recovery may be candidates for less frequent UDS testing[1].  Less frequent testing for appropriate patients will help to reduce the cost of these tests to the patient as well as the healthcare system and can reduce time away from work in patients in recovery who are employed[18].  A comprehensive economic analysis is beyond the scope of this study but an important area for future study.

Our study had several limitations. This is a retrospective descriptive study. As such, we did not have access to the patients’ medical charts and were unable to review the medication lists. Given this limitation, we were not able to confirm if the presence of benzodiazepines or the occasional opioids that were medically indicated. We may be managing a more stable panel of patients receiving MOUD, and our results may not be applicable to all primary care settings. Our providers had resources to easily refer unstable patients back to our ATP clinic for further management and this structure may not be available to providers in other practice locations. An unexpected UDS may or may not change a care plan for a patient and is likely to be clinician dependent but may alert a clinician that a patient may need a higher level of care than that of a primary care setting.

This descriptive study provides data on UDS results in patients being managed by their primary care physicians with buprenorphine-naloxone for MOUD. Given the relative stability of our particular office setting, these findings may represent a baseline expectation of the frequency of unexpected test results in this population. Further studies will be needed for correlation to patient medication lists. This information will help to further define best practices for patient-centered care of patients receiving buprenorphine-naloxone for recovery from OUD (Additional file 1).

## Conclusion

Patients being treated for opioid use disorder with buprenorphine-naloxone in an academic primary care spoke program had low rates of unexpected urine drug screens. Further research should be done on the frequency of testing and patient outcomes in this stable population in order to provide better guidelines on UDS monitoring in MOUD.

## Supplementary Information

**Additional file 1.** The opioid use disorder protocol at the author’s institution.

## Data Availability

The data set used for this project are available and accessible to the authors of this study.

## Abbreviations

ATP: Addiction Treatment Program
MOUD: Medication for opioid use disorder
PCPs: Primary care physicians
OUD: Opioid use disorder
UVMMC: University of Vermont Medical Center
UDS: Urine Drug Screen
