# Supplementary material for: A Descriptive analysis of urine drug screen results in patients with opioid use disorder managed in a primary care setting
Source: Addict Sci Clin Pract. 2021 Sep 30;16:59. doi: 10.1186/s13722-021-00264-4 (PMC8482571; doi:10.1186/s13722-021-00264-4)
Supplement: Supplementary file 1 — Additional file 1. The opioid use disorder protocol at the author’s institution. [file 13722_2021_264_MOESM1_ESM.docx]

| IDENT | MG7 |
| --- | --- |
| Type of Document | Policy |
| Applicability Type | Department-Level |
| Title of Owner | Dir UVM Medical Group |
| Title of Approving Official | VP Medical Group Operations |
| Date Effective | 5/4/2018 |
| Date of Next Review | 5/4/2021 |


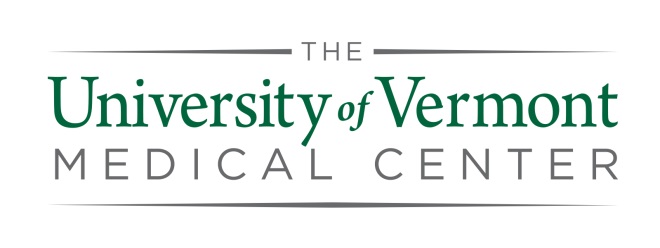


**TITLE**: Transition of Care - Primary Care and Addiction Treatment Program

**Purpose**: To provide a highly reliable process for patients transitioning from Chittenden Clinic (Hub) to the Patient Centered Medical Home (Primary Care), utilizing ATP to bridge transition from Chittenden Clinic to Primary Care.

**Procedure**: The following process will be utilized to guide the transition of a patient’s care from the Chittenden Clinic to ATP to Primary Care. In addition, the process details when it may be appropriate for a patient to return to ATP from Primary Care for additional support.

**Key Considerations for UVM MG PCP Patient Transition from Hub or Maternal Fetal Medicine (MFM) to Addiction Treatment Program**

- Patient has established with the UVM Medical Center for greater than 6 months from present date.
  - ATP Provider to validate with UVM Medical Center PCP office prior to accepting patient into ATP.
    - In addition, the availability of a suboxone prescriber within patient’s UVM Medical Center PCP practice will be verified.
  - *Exception:* Patient is an established patient of UVM Medical Center MFM and is transitioning to ATP from MFM (not from Hub). By PCP agreement, patient will establish care with a UVM Medical Center PCP as part of transition from MFM.
  - *Exception:* Patient does not have a PCP assigned or was not established at a minimum 6 months prior to current date - Through provider agreement, a UVM Medical Center PCP is identified and patient is transitioned to ATP from Hub.
  - *Exception:* Patient has a UVM MG PCP and Suboxone is prescribed externally. PCP may elect to transition patient to Addiction Treatment Program for on time evaluation/consultation prior to transition to PCP for prescribing. A referral will be placed to ATP within the electronic medical record.
- Number of patients managed by PCP will need to be verified to not exceed 30 patients per DEA requirements.
- Upon transition from Hub to ATP, a “Ready to Transition” visit will be scheduled with the UVM Medical Center prescriber to serve as a touch point for patient and provider.
  - Visit will be scheduled 5 weeks from start date of ATP management to reflect 4 week ATP transition.
  - If patient is not ready to successfully transition from ATP to PCP at 4 weeks, plan will be updated and scheduled appointment changed as appropriate.

**Key Considerations for Transition from ATP to PCP**

- Approved by ATP for transition
- “Ready to Transition” visit scheduled with prescriber no greater than one week post-discharge from ATP.
  - If patient has not seen PCP within 12 months, visit with PCP to be scheduled as well.
- Case review with MAT Team completed no less than 2 weeks in advance of discharge from ATP.
  - Scheduled visits with Primary Care MAT Team may occur separate from PCP and/or prescriber visit.
  - Weekly Care Coordination meeting will occur between ATP MAT and Primary Care MAT to discuss patient readiness and provide verbal handoff/coordination.
- A verbal handoff will occur between ATP provider and PCP/Prescriber. In addition, a Transitions of Care summary will be created and available in the electronic medical record.

**Key Considerations for Return to Day One from PCP**

- *See Appendix A*

**Management Within Primary Care**

***SEE APPENDIX A FOR CARE MANAGEMENT***

**Primary Care Treatment Agreement and Informed Consent (Appendix B)**

1. A new Primary Care Treatment Agreement and Informed Consent will be completed upon initiation of PCP care and scanned into the electronic medical record.
2. The ATPTreatment Agreement and Informed Consent will NOT be utilized in Primary Care as they have different provisions regarding confidentiality.

**Problem List**

1. If not already present on the patient’s problem list, F11.20 “Opioid Use Disorder” will be added upon transition to Primary Care by Primary Care MAT Team.
   1. If already present, Primary Care provider should mark as reviewed.
2. Prescriber of Suboxone and level of care to be noted in Overview section of problem using .OPIOIDREPLACEMENTPLAN (green, blue or gold).

**Health Maintenance**

1. If not already present on the patient’s Health Maintenance List, Opioid Replacement Management modifier will be manually added upon transition to Primary Care by Primary Care MAT Team.
2. When the modifier is added, the following plans and related topics are automatically added to the patient’s chart:
   1. Primary Care MAT Consent – Defaults to once and completed using Health Maintenance override.
   2. Primary Care MAT Agreement – Defaults to once and completed using Health Maintenance override.
   3. Pill Count – Defaults to weekly and automatically completed when pill count doc flow sheet row for number of pills remaining is populated.
   4. Prescription Screen (VPMS) – Defaults to weekly and automatically completed when the .VPMSQUERY smartpharase is completed.
   5. Urine Drug Screen – Defaults to weekly and automatically completed when internal Drug Screen 6 or internal Drug Screen Opiates Comprehensive orders are resulted or for external results, completed using Health Maintenance override.
   6. OBOT Stability Index – Defaults to monthly and completed using Health Maintenance override.
3. All topics will default in with a due date of the day the Health Maintenance Modifier was added.

**Drug Screens**

1. Observed drug screens should be completed externally to allow for appropriate observation process.
2. It is recommended all urine drug screen collections be observed and completed through Aspenti Lab or Dominion Labs, as an external order.
3. It is within provider discretion to request an unobserved drug screen collection or initiate an internal observed collection ensuring same sex staff/provider observer.
4. Standing order for a drug screen will be placed on the patient’s record upon transition to PCP by MAT Team.
   1. External Lab (Aspenti Lab or Dominion) will be notified by MAT Team to initiate faxed order for prescriber signature.
5. Point of Care Testing urine drug screen will not be utilized.

**VPMS Process**

1. VPMS process will be followed per State requirements.
2. Upon transfer from ATP to PCP, delegate will request access under prescriber, if not already in place.

**Monitoring Plan:**  Evaluate every 3 months for additions, changes and/or updates.

**dEFINITIONS:** N/A

**Related Policies/Procedures:**

State of Vermont VPMS Process

Department of Vermont Health Access Managed Care Entity Vermont Buprenorphine Practice Guidelines

**Appendix A: Levels of Patient Management**

**Appendix B: Prescription Agreement and Informed Consent**

**Appendix C: MAT Team Menu of Services**

**Reviewers:**

Robert Pierattini, MD Psychiatry Health Care Service Leader

Dawn Godaire, RN Director, Clinical Operations and Training

Claudia Berger, MD Medical Director, Adult Primary Care Burlington

John Chisholm, MD Physician, Family Medicine

Erica Gibson, MD Physician, University Pediatrics

Pam Farnham, RN Manager, Community Health Team

Bill Keithcart, MA, LADC Clinical Supervisor, ATP

Jennifer Parks Chief Compliance Officer

**OWNER:** Maureen Vinci, Dir UVM Medical Group

**APPROVING OFFICIAL:** Lisa Goodrich, VP Medical Group Operations

**APPENDIX A**

**Levels of Patient Management**

***Patient enters UVM MG PCP office from DayOne Suboxone Transition Program at Level Green or level otherwise determined by current clinical stability (Blue or Gold).***

|  | **Red** | **Yellow** | **Green** | **Blue** | **Gold** |
| --- | --- | --- | --- | --- | --- |
| **OBOT Stability Index Score** | HUB | Return To DayOne | Completion upon entry | Completion monthly | Completion as needed |
| **Drug Screen** | HUB | Return to DayOne | At least 1/week | At least 2/monthly | At least monthly |
| **Provider Appointments** | HUB | Return To DayOne | At least weekly | At least monthly | Monthly check in with MAT Team |
| **Script** | HUB | Return to DayOne | 28 day prescription cycle (14 day with 1 refill) | 28 day prescription cycle (14 day with 1 refill) | 28 day prescription cycle (14 day with 1 refill) |
| **Pill/Strip Counts** | HUB | Return To DayOne | At least monthly | As needed | As needed |
| **Care Coordinators**  **(MAT Team)** | HUB | Return to DayOne | At least weekly | At least monthly | At least monthly |
| **Minimum Time in Level** | n/a | Return to DayOne | Minimum 4 weeks | Minimum 4 weeks | n/a |

**Guidelines for Moving between Levels (and per provider discretion)**

1. Green to Blue
   1. Drug Screen showing presence of suboxone only x 2 months
   2. Attends 100% of appointments (bumps, provider cancellations and rescheduled appointments do not apply) x 2 months
   3. No failed strip counts x 2 months
   4. Compliant with expected behaviors
   5. Queries of VPMS show no evidence of unexplained, unadmitted or otherwise concerning provision of controlled substances
   6. OBOT Stability Index Score – No to all questions
2. Blue to Gold
3. Drug Screen showing presence of suboxone only x 6 months
4. Attends 100% of appointments (bumps, provider cancellations and rescheduled appointments do not apply) x 6 months
5. Compliant with expected behaviors
6. Queries of VPMS show no evidence of unexplained, unadmitted or otherwise concerning provision of controlled substances
7. OBOT Stability Index Score – No to all questions
8. Move to Level Yellow
9. Unapproved benzodiazepine use
10. Contraindicated ETOH use
11. Evidence of diversion
12. Increased frequency in script request or reports of missing scripts
13. Non-compliant with expected behaviors
14. Suspicious Drug Screen (stimulants, opiates and cocaine)
15. Two failed strip counts
16. Queries of VPMS show evidence of unexplained, unadmitted or otherwise concerning provision of controlled substances
17. 2 or more appointment no shows (bumps, cancellations and rescheduled appointments do not apply)
18. Adulterated UA or fail to complete random Urine Drug Screen or strip count
19. OBOT Stability Index Score – Yes to any of the questions
20. Move to Level Red
    1. Internal to DayOne with reassessment to return to Hub

**APPENDIX B**

**Office Based Medication Assisted Therapy (MAT)**

**Treatment Agreement for Buprenorphine Clients**

***FOR USE IN the MEDICAL HOME***

**Patient Name: ________________ DOB: __________ MRN: __________**

**In order to ensure the quality of your care and to increase success in recovery, we ask you to agree to the following treatment agreement/compliance form.**

**Please read carefully and fill in the blanks and/or initial.**

1. I agree to have a working telephone and keep providers updated with any changes. My current phone number is: ____________________.
2. I authorize release of information and agree to sign all necessary releases to ensure collaboration of care with all physician and/or counselors involved in my treatment: __________.
3. In order to promote a therapeutic relationship, we (care team members and patient) agree to treat each other with mutual respect and in the appropriate manner.
4. I agree to use a single pharmacy for *all* controlled substances, including buprenorphine. My current pharmacy is: ____________________.
5. I agree to use a single physician to prescribe my medication assisted therapy (MAT): __________.
6. I understand that prescription (buprenorphine) refills are **my** responsibility. I agree to give **at least 48 hours notice** for needed refills, taking holidays and weekends into consideration: __________.
7. I agree to take my medications as the doctor and other treatment staff have instructed and not to alter the way I take my medication without first consulting the doctor: ________.
8. I agree that the medication I receive is my responsibility and that I will keep it in a safe, secure place. I agree to keep my medication in a lock box/lock bag/safe. Lost medications will be addressed by the treatment team who will review the situation and come up with a treatment plan: ________.
9. I agree to maintain and if needed revise a treatment plan with my primary care provider and that the plan is shared with my physician(s) and any other providers that are pertinent to my recovery. The purpose of this is to ensure that all parties are working in collaboration to provide the best treatment possible: __________.
10. I agree to attend, and be on time for, my appointments with both my provider and primary care medication assisted treatment team as determined by my treatment plan__________.
11. I understand that compliance with all of my medication is expected, unless a change is made by my prescribing physician. If a change is made, my prescribing physician and I will notify the other professionals involved with my care: __________.
12. I agree to inform all of my professional providers about any of the following:
13. Relapse: __________.
14. Any change in prescription or use: __________.
15. Any change in prescribing physician or PCP: __________.
16. I understand that Urine Drug Testing (UDS) will be conducted randomly, observed and the results will be shared with the appropriate professionals

in my care. I agree to provide a urine sample within 24 hours of being called: __________.

1. I agree to random film/pill counts within 24 hours of being called:__________.
2. I agree to not eat any foods or bakery items that contain **poppy seeds**. I agree to not use any **mouth wash or cough syrup** containing alcohol as these can result in positive drug screens: __________.
3. I understand the following indicates non-compliance with the MAT program:
4. Failure to provide a urine drug screen at physician/MAT Team

request: __________.

1. Presence of non-prescribed substances in urine screens: __________.
2. Abnormal temperature range of urine drug screen: __________.
3. Falsification of a urine drug screen: __________.
4. Failure to attend medical and counseling appointments: __________.
5. Arriving to appointments under the influence of drugs or alcohol: ____ .
6. Diverting medication: _________.
7. I understand that buprenorphine will be present on my medication list and “opioid use disorder” will be present on my problem list in the UVM Medical Center electronic medical record __________.
8. I understand that medication alone is not sufficient treatment for my disease. Counseling requirements will be at the discretion of the MAT Team, including the prescribing doctor: ________.

**I understand that non-compliance will be discussed with my physician and clinical team and may result in a change of treatment plan, a behavioral contract, or a possible discharge from the MAT program:** __________**.**

**Patient Name (please print):** ____________________ **Date:**  ___________________

**Patient Signature:** _____________________________________________________

**MAT Team Signature and title:** __________________________________________

**Primary Care Provider Signature and title:** _________________________________

**Date of Transition to PCP:** _________________

**Office Based Medication Assisted Therapy (MAT)**

**Patient Consent for Buprenorphine Clients**

***FOR USE IN PRIMARY CARE***

**Patient Name:** ____________________________ **MRN:** __________________

**Consent for Treatment with Suboxone**

- Suboxone is an FDA approved medication for treatment of people with heroin or other opioid addiction. Suboxone can be used for detoxification or for maintenance therapy. Maintenance therapy can continue as long as medically necessary. There are other treatments for opiate addiction, including methadone, naltrexone, and some treatments without medications that include counseling, groups and meetings.
- After you become stabilized on Suboxone, it is expected that other opioids will have less of an effect. Attempts to override the Suboxone by taking more opioids could result in an opiate overdose. You should not take any other medications without discussing it with your physician first.
- Combining Suboxone with alcohol or other sedating medications is dangerous. The combination of Suboxone with benzodiazepines (such as Valium, Librium, Ativan, Xanax, Klonopin, etc.) has resulted in death.
- Suboxone is a combination of buprenorphine with a short acting opioid blocker (naloxone). Buprenorphine will maintain your physical dependence on opioids and if you discontinue it suddenly, you will likely experience withdrawal.
- Suboxone film or tablets must be held under the tongue until they dissolve completely. It is important not to talk or swallow until the film or tablet dissolves. This takes up to ten minutes. Suboxone is absorbed over the next 30 – 120 minutes from the tissue under the tongue. Suboxone is poorly absorbed from the stomach. If you swallow the tablet, you will not have the important benefits of the medication and it may not relieve your withdrawal.
- You and your doctor will determine the appropriate dose, which will vary from one person to another. The effects of buprenorphine plateau above a certain dose, so higher doses do not necessarily offer more benefit.

I have read and understand these details about Suboxone treatment.

**Patient Name (please print):** ____________________ Date**:** ___________________

**Patient Signature:** _____________________________________________________

**Primary Care Provider Signature and title:** _________________________________

Date of Transition to PCP: _________________

**Office Based Medication Assisted Therapy (MAT)**

**Treatment Agreement for Buprenorphine Clients**

***FOR USE IN DAYONE SUBOXONE TRANSITION PROGRAM***

**Patient Name:** ____________________________ **MRN:** __________________

**In order to ensure the quality of your care and to increase success in recovery, we ask you to agree to the following treatment agreement/compliance form.**

**Please read carefully and fill in the blanks and/or initial.**

1. I agree to have a working telephone and keep providers updated with any changes. My current phone number is: ____________________.
2. I agree to sign all necessary releases to ensure collaboration of care with all physician and/or counselors involved in my treatment: __________.
3. I agree to use a single pharmacy for *all* my prescriptions, specifically all prescribed medications. My current pharmacy is: ____________________.
4. I agree to use a single physician to prescribe my medication assisted therapy (MAT): __________.
5. I agree to develop a treatment plan with my primary therapist and that the plan is shared with my physician(s) and any other providers that are pertinent to my recovery. The purpose of this is to ensure that all parties are working in collaboration to provide the best treatment possible: __________.
6. I agree to attend treatment sessions, both group and individual as determined by my treatment plan, unless I have an excused absence and my prescribing physician is aware of the absence: __________.
7. I understand that compliance with all of my medication is expected, unless a change is made by my prescribing physician. If a change is made, my prescribing physician and I will notify the other professionals involved with my care: __________.
8. I agree to inform all of my professional providers about any of the following:
9. Relapse: __________.
10. Any change in prescription or use: __________.
11. Any change in prescribing physician: __________.
12. I understand that Urine Drug Testing (UDS) will be conducted randomly, observed and the results will be shared with the appropriate professionals

in my care. I agree to provide a urine sample within 24 hours of being called: __________.

1. I agree to random film/pill counts within 24 hours of being called: __________.
2. I agree to not eat any foods or bakery items that contain **poppy seeds**. In addition I agree to not use any **mouth wash** or cough syrup containing alcohol as these can result in positive drug screens: _________________.
3. I understand the following may indicate non-compliance with the MAT program:
4. Failure to produce a urine drug screen at physician/counselor

request: __________.

1. Presence of non-prescribed substances in urine screens: __________.
2. Abnormal temperature range of urine drug screen: __________.
3. Falsification of a urine drug screen: __________.
4. Aggressive behavior toward staff: __________.
5. Failure to attend medical and counseling appointments as

scheduled: __________.

1. Medication diversion: __________.
2. I understand that suboxone will be present on my medication list and “opioid use disorder” will be present on my problem list in the UVM Medical Center electronic health record.

**I understand that non-compliance will be discussed with my physician and clinical team and may result in a change of treatment plan, a behavioral contract, or a possible discharge from the MAT program:** __________**.**

**Patient Name (please print):** ____________________ Date**:** ___________________

**Patient Signature:** _____________________________________________________

**DayOne Provider Signature and title:** _____________________________________

**Office Based Medication Assisted Therapy (MAT)**

**Patient Consent for Buprenorphine Clients**

***FOR USE IN DAYONE SUBOXONE TRANSITION PROGRAM***

**Patient Name:** ____________________________ **MRN:** __________________

**Consent for Treatment with Suboxone**

- Suboxone is an FDA approved medication for treatment of people with heroin or other opioid addiction. Suboxone can be used for detoxification or for maintenance therapy. Maintenance therapy can continue as long as medically necessary. There are other treatments for opiate addiction, including methadone, naltrexone, and some treatments without medications that include counseling, groups and meetings.
- After you become stabilized on Suboxone, it is expected that other opioids will have less of an effect. Attempts to override the Suboxone by taking more opioids could result in an opiate overdose. You should not take any other medications without discussing it with your physician first.
- Combining Suboxone with alcohol or other sedating medications is dangerous. The combination of Suboxone with benzodiazepines (such as Valium, Librium, Ativan, Xanax, Klonopin, etc.) has resulted in death.
- Suboxone is a combination of buprenorphine with a short acting opioid blocker (naloxone). Buprenorphine will maintain your physical dependence on opioids and if you discontinue it suddenly, you will likely experience withdrawal.
- Suboxone film or tablets must be held under the tongue until they dissolve completely. It is important not to talk or swallow until the film or tablet dissolves. This takes up to ten minutes. Suboxone is absorbed over the next 30 – 120 minutes from the tissue under the tongue. Suboxone is poorly absorbed from the stomach. If you swallow the tablet, you will not have the important benefits of the medication and it may not relieve your withdrawal.
- You and your doctor will determine the appropriate dose, which will vary from one person to another. The effects of buprenorphine plateau above a certain dose, so higher doses do not necessarily offer more benefit.

**Authorization to disclose Health Information including Alcohol and Substance Abuse Treatment Information**

- I understand that my medical records related to the DayOne Program have confidentiality protections under federal law. I authorize the DayOne Program to disclose information regarding my treatment and participation in the DayOne Program, including but not limited to dates of service, medications, diagnoses, laboratory tests, and medical and social history, to The University of Vermont Medical Center and its staff and providers, for the purpose of treatment, coordination of care, and administration of the DayOne Program. This authorization includes authorization for the DayOne Program and The University of Vermont Medical Center to re-disclose my DayOne Program records to my Primary Care Physician for the purpose of treatment and coordination of my care. Initials: ________
- I understand that my DayOne Program records will be stored in the University of Vermont Medical Center’s electronic health record and that information about my diagnosis of opioid use disorder and medications such as Suboxone may be viewable by the University of Vermont Medical Center, its staff, and others who are involved in my medical care. I authorize those involved in my care to view this information. Initials: ________
- I understand that I have the right to revoke this authorization for the disclosure of my DayOne Program records except and to the extent that action has been taken in reliance on it. This authorization will remain in effect until I revoke it by notifying the DayOne Program in writing. Initials: _________

I have read and understand these details about Suboxone treatment and disclosure of my protected health information.

**Patient Name (please print):** ____________________ Date**:** ___________________

**Patient Signature:** _____________________________________________________

**DayOne Provider Signature and title:** _____________________________________

**APPENDIX C**

Menu of services:

Day One- DO

MAT team- MAT

1. **Comprehensive Care Management:**

- Outreach to patients who are lost to contact: home visits or alternate visit site, evening phone calls, letters- MAT
- Assessment of preliminary service needs: Intake paperwork assessment which addresses general health needs/interests, mental health and addiction history.- DO
- Brief therapy- DO and MAT
- SBIRT: Screening, Brief Intervention and Referral to Treatment- DO and MAT

1. **Care Coordination**

- UDS – random observed: monthly, bi monthly, weekly- DO and MAT
- Strip Counts: as above- MAT
- VPMS checks (usually monthly or quarterly)-DO and MAT
- Monitor treatment progress through monthly contact with therapist- DO and MAT
- Case management for access to social, vocational and substance abuse supports in the community-DO and MAT

1. **Health Promotion**

- Health education including nutritional counseling, medication effects, chronic health care issues and opioid dependence and treatment- MAT
- Motivational interviewing for to engage patient in healthy life-style-MAT

1. **Comprehensive Transitional Care**

- Case management for access to other health providers or social services: Referral to Intensive Outpatient Program- MAT

1. **Individual and Family Support**

- Providing outreach and brief counseling DO and MAT
- Providing information about community services- DO and MAT
- Assist with navigating health care system-MAT

1. **Referral to Community and Social Support Services**

- Maintain up-to-date information about community resources: food shelf, housing, health insurance- MAT
- Provide information to patient as needed- MAT
